# The Importance of Serum N-Terminal Pro-Brain Natriuretic Peptide and Endogenous Hydrogen Sulfide for Predicting Coronary Artery Lesions in Pediatric Kawasaki Disease Patients: Findings From a Tertiary Care Hospital in Karachi, Pakistan

**DOI:** 10.7759/cureus.9016

**Published:** 2020-07-06

**Authors:** Muhammad Bilal, Abdul Haseeb, Alina Saeed, Muhammad Ahad Sher Khan

**Affiliations:** 1 Internal Medicine-Pediatrics, Dr. Ruth K. M. Pfau, Civil Hospital, Karachi, PAK; 2 Internal Medicine, The Wright Center for Graduate Medical Education, Scranton, USA; 3 Internal Medicine, Ziauddin University, Karachi, PAK; 4 Internal Medicine, Dr. Ruth K. M. Pfau, Civil Hospital, Karachi, PAK

**Keywords:** kawasaki disease (kd), coronary artery lesion (cal), serum nt-probnp, h2s, il-6

## Abstract

Introduction

Kawasaki disease (KD) is an idiopathic, acute systemic vasculitis typically affecting medium-sized blood vessels with an inclination towards the coronary arteries. There is no specific diagnostic test established for it yet. Hence, our study aims to evaluate serum N-terminal pro-brain natriuretic peptide (NT-proBNP), hydrogen sulfide (H_2_S), and interleukin-6 (IL-6) levels as potential diagnostic tools in children with KD and determine its relationship with the development of coronary artery lesions (CAL) in the pediatric population visiting a tertiary care hospital in Karachi.

Methods

A prospective observational study was performed on a sample of 500 children at a tertiary care hospital over a period of two years from June 2017 to June 2019. Blood samples were collected from two groups labeled CAL and non-coronary artery lesion (NCAL), and different biomarkers including NT-proBNP, IL-6, and H_2_S were compared between them to predict the diagnostic properties of each marker.

Results

Among the 500 children, 50% were between the age of one to five years. All presented with fever and varying degrees of associated symptoms. On lab investigations, levels of NT-proBNP and IL-6 during the acute phase of the disease were found to be higher in the CAL group than the NCAL and control groups (p<0.001). However, H_2_S levels during the acute attack were significantly lower in the CAL group when compared to the NCAL or control groups (p<0.001).

Conclusion

Elevated levels of NT-proBNP and IL-6 can be utilized as potential clinical markers for identifying children at risk of developing CAL as a complication of KD. Reduced H_2_S levels are also proposed as an indicator of progress towards CAL and should be considered in reaching a diagnosis.

## Introduction

Kawasaki disease (KD) is an idiopathic, acute systemic vasculitis. It generally affects medium-sized blood vessels and is seen to have an inclination towards the coronary arteries. The condition is now considered to be the leading cause of acquired cardiac defects in children in developed countries [1]. It is a childhood disease with most patients under five years of age, and children aged <6 months have a higher risk of cardiac complications secondary to KD [2]. It classically presents with persistent high-grade fever and four out of the five of the following clinical features: bilateral conjunctival redness, erythematous oral mucosa, polymorphous rash, lesions on extremities, and cervical lymphadenopathy [3].

Since the condition is idiopathic, there is no specific diagnostic test established for it yet. Around 30-50% of KD patients, who do not receive treatment, develop transient coronary artery dilation, and one-fourth of them progress to have serious complications, with coronary artery lesion (CAL) being the most common. Other complications include myocardial infarction (MI), coronary artery stenosis, or even sudden death [4-5]. Hence, the importance of timely diagnosis and prompt management cannot be overstated. Moreover, since the resulting cardiac vascular lesions show inflammation and necrosis of blood vessels, multiple inflammatory mediators and biological molecules have been studied in recent years to help the clinicians in reaching a diagnosis [3]. Among them, pro-brain natriuretic peptide (proBNP) has been proposed to be linked to coronary artery dilation in children with KD and has gained good diagnostic value in cases of high suspicion [6]. Ventricular myocytes release BNP and its inactivated form N-terminal proBNP (NT-proBNP) when the cardiac wall undergoes excessive stretch and ischemia. Therefore, they are frequently used as a clinical marker in assessing congestive heart failure and coronary artery diseases in adults and may also prove to be beneficial in KD [7]. Interleukin-6 (IL-6) is a pro-inflammatory marker that plays an important role in the development of CAL in KD. Serum hydrogen sulfide (H_2_S) is a cardioprotective intrinsic signaling molecule that is produced by the blood vessels and is found to be low in KD patients who develop CAL [8]. However, a paucity of data has prevented them from being used as diagnostic markers in the KD attack. 

In Pakistan, limited data is available regarding the prevalence of KD and the effectiveness of serum markers in its diagnosis. However, Pakistan lies in a region where the estimated incidence of KD is about 70 per 100,000 children, which is a significant statistic, and it underlines the importance of having an effective diagnostic tool [9]. Furthermore, local studies have reported a high incidence of coronary artery involvement in children with KD (about 41% in Karachi and 32% in Islamabad) [10,11]. Therefore, early identification of these children becomes essential; hence, our study aimed to evaluate serum NT-proBNP, H_2_S, and IL-6 levels in children suffering from KD and determine their association with the development of CAL in the pediatric population visiting a tertiary care hospital in Karachi.

## Materials and methods

This prospective observational study was carried out at a tertiary care hospital in Karachi over a period of two years from June 2017 to June 2019. The study was approved by the ethical review board of the Dow University of Health Sciences. Informed consent in written form was obtained from the parents of all children included in the study.

Five hundred children aged between two months and 12 years who were admitted to the pediatric department during our study period with a diagnosis of KD were included in the study. Children with comorbidities, underlying heart diseases, chronic illnesses, or other confirmed diagnoses for their fever and associated symptoms were excluded from the study. In all patients, the diagnosis of KD was made using the criteria set by the American Heart Association [12]. Moreover, each child diagnosed with KD was assessed for the physical symptoms of fever, redness of eyes, oral mucosal changes, changes in the extremities, rash, and other manifestations. On admission, blood samples were collected from each patient and sent for complete blood count (CBC), C-reactive protein (CRP), erythrocyte sedimentation rate (ESR), alanine transaminase (ALT), electrolytes, and serum albumin level. The levels of serum biomarkers NT-proBNP, H_2_S, and IL-6 were also tested in each patient during the acute phase of the disease. Moreover, 2 ml of blood samples were collected before starting treatment for cases and control groups. The samples collected were kept at room temperature for half an hour and later centrifuged for 10 minutes at 4 °C at the speed of 3,000 r/min. The supernatant was separated by pouring it off and was kept at -70 °C before performing quantitative serum analysis for biomarkers.

Before starting the treatment, all children were screened for coronary artery aneurysm by a pediatric cardiologist with GE Vivid 7 (GE Healthcare, Chicago, IL) ultrasound machine and Philips Sonos 5500 (Philips Healthcare, Best, Netherlands) echocardiographic machine. The 7 Hz probe was used for younger children and a standard 3 Hz probe for older ones. Those identified with CALs were included in the CAL group while others were labeled as the non-coronary artery lesion (NCAL) group. All patients were followed through the course of the disease and were given appropriate treatment. The test for biomarker levels was repeated after 15 days from the initial presentation by taking blood samples from children visiting the outpatient department for follow-up and also from those who remained admitted. Laboratory values were recorded in their respective files. The data collected over time was compiled from records for analysis. SPSS Statistics (IBM, Armonk, NY) was used for analysis and different tests were applied for comparing quantitative data. Associations were made for the qualitative data and were tabulated.

## Results

Our study included a total of 500 children who presented with KD. A summary of the clinical features of the patients is presented in Table 1. Of the 500, 50 were below the age of one year, 250 were between one to five years of age, 150 were between 5-10 years, and 50 were between 10-12 years. All of them presented with a fever as it is the cardinal sign of KD. Other symptoms mentioned earlier varied in their presentation. The majority (475) of the children had redness in the eyes; 400 had oropharyngeal changes, while 375 children had swelling of hands and feet. On examination, the other commonly present symptoms were cervical lymphadenopathy (325), rash (300), hepatomegaly (275), joint pain (265), sensorineural hearing loss (200), and urethritis (150).

**Table 1:** Clinical features of patients with KD KD: Kawasaki disease

Variable	Number of cases (%); N=500
Age, years	
<1	50 (10)
1-5	250 (50)
5-10	150 (30)
10-12	50 (10)
Symptoms	
Fever	500 (100)
Redness in the eye	475 (95)
Changes on the lips and in the oral cavity	400 (80)
Swelling of hands	425 (85)
Swelling of feet	350 (70)
Swelling of hands and feet	375 (75)
Rashes	300 (60)
Cervical lymphadenopathy	325 (65)
Hepatomegaly	275 (55)
Joint pain	265 (53)
Sensorineural hearing loss	200 (40)
Urethritis	150 (30)

Table 2 lists the different abnormalities detected on the lab tests. Increased CRP and ESR were the most common findings present; they were found in 80% of the patients. Leukocytosis (60%), thrombocytosis (70%), and raised ALT levels (70%) were some of the other common findings. However, hyponatremia (20%) and hypoalbuminemia (16%) were not frequently observed.

**Table 2:** Laboratory test results of patients with KD KD: Kawasaki disease; CRP: C-reactive protein; ESR: erythrocyte sedimentation rate; ALT: alanine transaminase

Variable	Number of cases (%); N=500
Leucocytosis	300 (60)
Thrombocytosis	350 (70)
Increased CRP	400 (80)
Increased ESR	400 (80)
Raised ALT	350 (70)
Hyponatremia	100 (20)
Hypoalbuminemia	80 (16)

As shown in Table 3 below, levels of NT-proBNP and IL-6 during the acute phase of the disease were found to be higher in the CAL group than the NCAL and control groups (p<0.001). The control sample included 50 children with febrile illnesses of variable causes. However, H_2_S levels during the acute attack were significantly lower in the CAL group when compared to the NCAL or control groups (p<0.001).

**Table 3:** Comparison of NT-proBNP, H2S, and IL-6 levels in the CAL group, NCAL group, and control group in the acute phase NT-proBNP: N-terminal pro-brain natriuretic peptide; CAL: coronary artery lesion; NCAL: non-coronary artery lesion; H_2_S: hydrogen sulfide; IL-6: interleukin-6; SD: standard deviation

Group	Number of patients	NT-proBNP, mg/L, mean ± SD	H_2_S, mmol/L, mean ± SD	IL-6, ng/L, mean ± SD
CAL	300	1.89 ± 0.33	27.98 ± 7.1	149.02 ± 21.2
NCAL	200	0.88 ± -0.21	38.99 ± 9.1	119.43 ± 13.2
Control	50	0.32 ± 0.14	70.71 ± 13.1	68.43 ± 9.1
P-value		<0.001	<0.001	<0.001

Table 4 shows the values of biomarkers during the recovery phase, which was two weeks after the initial presentation with fever. In the recovery phase, the levels of NT-proBNP and IL-6 remained significantly higher in the CAL group than the control group (p<0.001). Similarly, the H_2_S levels in the CAL group continued to be lower than the controls (p<0.001).

**Table 4:** Comparison of NT-proBNP, H2S, and IL-6 levels in the CAL group, NCAL group, and control group in the recovery phase NT-proBNP: N-terminal pro-brain natriuretic peptide; CAL: coronary artery lesion; NCAL: non-coronary artery lesion; H_2_S: hydrogen sulfide; IL-6: interleukin-6; SD: standard deviation

Group	Number of patients	NT-proBNP, mg/L, mean ± SD	H_2_S, mmol/L, mean ± SD	IL-6, ng/L, mean ± SD
CAL	300	1.21 ± 0.23	37.96 ± 9.1	99.32 ± 21.2
NCAL	200	0.59 ± -0.11	51.79 ± 6.1	90.63 ± 16.2
Control	50	0.32 ± 0.14	70.71 ± 13.1	68.43 ± 9.1
P-value		<0.001	<0.001	<0.001

## Discussion

KD is an idiopathic condition. While it is generally believed that an underlying infectious agent causes the symptoms of KD in a genetically predisposed individual, not much is known about its pathogenesis [13]. Clinically, it is seen as an inflammatory condition that typically affects the heart, causing serious complications in predisposed children. Hence, it is critical to promptly diagnose the condition and provide appropriate treatment in order to eliminate the complications. However, the greatest hurdle in starting timely treatment is the diagnosis itself. Due to the uncertain pathophysiology of KD, a definite mode for its diagnosis has not been established yet. Hence, the clinicians currently rely on the duration of fever (i.e, five days) and other additional symptoms to diagnose a case of KD. This delays management and increases the risk of morbidity in children.

In recent years, multiple biomarkers have been studied as part of an attempt to establish a diagnostic tool. Among these, NT-proBNP has gained recognition as a potential marker and can be utilized in excluding KD in children with fever of unknown origin [14,15]. BNP and its inactive byproduct, NT-proBNP, are formed from pre-proBNPs that are released by the ventricular myocytes in response to stretch, ischemia, and inflammation [16]. In KD, left ventricular dysfunction causes the release of BNP; hence, it can be a useful marker for identifying CAL [17]. The pancarditis occurring in KD and the rise in BNP in response to inflammation further supports this link [12]. In our study, we found significantly increased levels of NT-proBNP in children who developed CAL as compared to the NCAL and control groups. A study by Kaneko K et al. has also reported similar findings [18]. Furthermore, the acute inflammatory process activates macrophages that release cytokines including IL-6 and tumor necrosis factor-alpha (TNF-alpha), resulting in the progression of CAL and the associated symptoms. We found a significant rise in the levels of IL-6 among children with CAL versus the NCAL and control groups. One specific study has shown a significantly greater rise in IL-6 in children who developed CAL as compared to other inflammatory cytokines [19]. Moreover, during the recovery phase of the disease, these markers remained significantly raised as compared to the controls, indicating their role in the pathogenesis of KD.

H_2_S is a protective endogenous mediator released by endothelial and smooth muscle cells [20]. It is known that multiplication of vascular smooth muscle cells and intercellular adhesions are decreased by H_2_S, thereby preventing the formation of vascular lesions including atherosclerosis [21,22]. Since H_2_S can also lead to an enhanced recovery in ischemic or reperfused myocardial tissue by preserving adenosine triphosphate (ATP) pools in cells, its low levels can lead to the destruction seen in inflamed CALs [23]. This suggests that decreased H_2_S levels may be involved in the pathogenesis of KD as found in our study. We found significantly decreased levels of H_2_S in children who developed CAL as compared to the NCAL and control groups. Early detection of the reduction in H_2_S levels can, therefore, be utilized in identifying children at risk of developing CAL.

## Conclusions

Elevated levels of NT-proBNP and IL-6 can be utilized as potential clinical markers for identifying children at risk of developing CAL as a complication of KD. This could help in the timely management of the patients and reducing lifelong morbidities associated with the condition. Reduced H_2_S levels are also proposed as an indicator of progress towards CAL and should be considered in reaching a diagnosis.
